# Fibrosis independent atrial fibrillation in older patients is driven by substrate leukocyte infiltration: diagnostic and prognostic implications to patients undergoing cardiac surgery

**DOI:** 10.1186/s12967-019-02162-5

**Published:** 2019-12-10

**Authors:** Christie M. Aguiar, Kareem Gawdat, Stephanie Legere, Jean Marshall, Ansar Hassan, Petra C. Kienesberger, Thomas Pulinilkunnil, Mathieu Castonguay, Keith R. Brunt, Jean-Francois Legare

**Affiliations:** 1 IMPART Investigator Team Canada (https://impart.team/), Saint John, NB Canada; 2 Dalhousie Medicine New Brunswick, Saint John, NB Canada; 3 Department of Microbiology and Immunology, Dalhousie University, Halifax, NS Canada; 4 Department of Biochemistry & Molecular Biology, Dalhousie University, Halifax, NS Canada; 5 Department of Pathology, Dalhousie University, Halifax, NS Canada; 6 New Brunswick Heart Centre, 400 University Av, PO Box 2100, Saint John, NB E2L 4L2 Canada

**Keywords:** Atrial fibrillation, Cytokines, Macrophages (MoΦ), Human atrium, Cardiac surgery, Leucocyte infiltration, Inflammation, Cardiac fibrosis, NLR

## Abstract

**Background:**

The objectives of the study were to characterize and quantify cellular inflammation and structural remodeling of human atria and correlate findings with molecular markers of inflammation and patient surrogate outcome.

**Methods:**

Voluntary participants undergoing heart surgery were enrolled in the study and blood samples were collected prior to surgery, and right atrium samples were harvested intraoperatively. Blood samples were analyzed by flow cytometry and complete blood counts. Atrial samples were divided for fixed fibrosis analysis, homogenized for cytokine analysis and digested for single cell suspension flow cytometry.

**Results:**

A total of 18 patients were enrolled and samples assessed. Isolated cells from the atria revealed a CD45+ population of ~ 20%, confirming a large number of leukocytes. Further characterization revealed this population as 57% lymphocytes and 26% monocyte/macrophages (MoΦ), with the majority of the latter cells being classical (CD14++/CD16−). Interstitial fibrosis was present in 87% of samples and correlated significantly with patient age. Older patients (> 65) had significantly more atrial fibrosis and cellular inflammation. AFib patients had no distinguishing feature of atrial fibrosis and had significantly greater CD45+ MoΦ, increased expression of MMP9 and presented with a significant correlation in length of stay to CCL-2/MCP-1 and NLR (neutrophil-to-lymphocyte ratio).

**Conclusion:**

Atrial fibrosis is correlated with age and not determinate to AFib. However, severity of atrial leukocyte infiltration and markers of matrix degradation are determinant to AFib. This also correlated with CCL2 (or MCP-1) and NLR-indicative of marked inflammation. These data show the potential importance of diagnostic and prognostic assessments that could inform clinical decision making in regard to the intensity of AFib patient management.

## Background

Atrial Fibrillation (AFib) is an irregular heart rhythm that affects ~ 1.5% of the population globally (~ 10% adults over 80 years of age), can lead to heart failure or stroke, and is a major determinant in patient quality of life [1, 2]. AFib may be asymptomatic or manifest various symptoms such as palpitations, fatigue, shortness of breath, dizziness, and chest pain [3]. AFib is currently incurable, and medical management seeks to reduce AFib events pharmacologically (ex. dofetilide) through rate and rhythm control strategies. This approach has limited long-term utility, and additional therapeutic modalities require ablative or surgical maze procedures to physically redirect/control electrical current flow. However, these approaches can also fail to fully resolve AFib. The ultimate goal of treatment is to reduce the likelihood of more catastrophic events (ex. thromboembolic stroke) by using anticoagulation therapy, and to reduce myocardial demand (ex. beta blockers).

Risk factors for AFib include conditions that cause atrial overload and stretch, such as hypertension, valvular heart disease, pulmonary congestion, and coronary artery disease [4]. Non cardiac risk factors are advanced age, obesity, metabolic syndrome, obstructive sleep apnea, kidney disease, and smoking [5]. The specific mechanisms of AFib are not clearly defined, as there is a complex etiology of structural remodelling, and contractile and electrophysiological abnormalities that promote the onset and perpetuation of AFib [6]. AFib has also been associated with inflammatory conditions such as myocarditis and pericarditis and iatrogenically induced by cardiac surgery. Inflammation has been the underlying factor in several cardiovascular diseases and AFib is comorbid with hypertension, coronary artery disease, and heart failure [7]. Recent reports have linked AFib to the presence of inflammation associated with serum levels of pro-inflammatory biomarkers [8]. Mechanisms associated with AFib include oxidative stress, fibrosis, thromboembolism, endothelial dysfunction, adhesion and invasion of inflammatory cells, and secretion of cytokines as the major cellular and molecular sequalae contributing to the progression of the hypercoagulable state of AFib. Cytokines, such as TNF-α, CRP, IL-6, IL-8 and IL-10, are some of the biomarkers found to be elevated in AFib [9], as are increased populations of neutrophils and lymphocytes proportionately (NLR ratio) [10] in AFib patients compared to those in sinus rhythm (SR) [11]. Thus the “AFib begets AFib” phenomenon continues, but it remains to be determined whether inflammation is the incipient cause, consequence, or both with respect to AFib pathophysiology and thus clinical outcome.

Work from our group and others have shown that cellular immunity and inflammation plays a key role in myocardial disease, and is fundamental to our understanding of myocardial fibrosis, a common pathological feature of patients with heart disease [10, 12, 13]. Previous investigators have suggested that a significant amount of atrial fibrosis can be seen, and appears to correlate with inflammation and the presence of AFib [14–17]. Similarly, there is evidence that fibrosis increases with age and that age is a risk factor for AFib, but its relationship to inflammation is unclear [18, 19]. In this study we examine the link between inflammation and AFib by studying inflammatory cells in the human atrium that are determinant to development of AFib.

The objectives of the present investigation were to characterize and quantify cellular inflammation and structural remodeling of the atria and correlate findings with molecular markers of inflammation and patient surrogate outcome. Here we characterize the inflammatory burden seen in atrial tissue obtained from patients undergoing cardiac surgery by focusing on immune cells and their related cytokines using novel cell isolation and purification techniques, secreted protein analysis, and flow cytometry.

## Methods

### Study population

Voluntarily consenting patients undergoing non-emergent heart surgery at the QEII Health Science Centre (Halifax) were prospectively enrolled. Informed consent was obtained for all participating patients and all patients received standard of care treatment. The present investigation was part of a larger research program called Restitution Enhancement in Arthritis and Chronic Heart disease (REACH), which facilitated the collection and processing of tissue for bio-banking from patients undergoing heart surgery.

### Research ethics

This study was approved by the institutional Research Ethics Board (Nova Scotia Health Authority). All personal identifiers were stripped prior to data analysis to ensure patient anonymity and confidentiality.

### Study procedures

In all patients, blood samples were collected prior to skin incision and the atrial samples were taken at the time of cannulation. Atrial tissue samples were divided into various pieces for multiple immediate and future analyses: (1) cellular isolation, (2) histology, including immunohistochemistry, (3) RNA/protein.

Cardiac surgery was performed with cardiopulmonary bypass and anticoagulation was achieved using intravenous heparin given at a dose of 400 IU/kg with a target activated clotting time (ACT) greater than 450 s. Antifibrinolytic agents were given to all patients and consisted mainly of tranexamic acid. Intermittent cold blood cardioplegia was delivered in an antegrade or retrograde fashion based on surgeon preference. Protamine sulfate was given for reversal of heparin in all patients.

Resumption of routine postoperative medications occurred as indicated and included anti-platelet agents within 24 h, statins, and β-blockers. All patients were monitored in the cardiac intensive care unit for a minimum of 24 h and an intermediate care unit for an additional 24 h. Therefore a minimum of 48 h of continuous cardiac monitoring was used in all patients to detect AFib. New AFib was defined according to the Society of Thoracic Surgeons definition as any episode of AFib occurring in-hospital after CABG surgery that required intervention (β-blockers, calcium channel blockers, amiodarone, anticoagulation, or cardioversion) [20].

### Variable selection

Preoperative clinical characteristics of interest included age, sex, New York Heart Association (NYHA) functional class, urgency of surgery (urgent if required within 24 h, in-hospital urgent if the patient required hospitalization until the time of surgery, and elective or outpatient) and diabetes. Intraoperative variables included pump time and clamp time. Outcome variables included in-hospital mortality, atrial fibrillation, stroke, and length of hospitalization (length of stay; LOS).

### Atrial tissue processing

Atrial samples were processed immediately for the purposes of cellular isolation for flow cytometry. Tissue samples were finely minced with scalpel blades, followed by mechanical and enzymatic digestion using a cocktail of enzymes consisting of Collagenase II (1 mg/mL, Worthington Biochemical Corp., Lakewood, NJ, USA) and dispase (2 mg/mL) in serum free Hank’s Balanced Salt Solution (HBSS, 2 mmol/L l-glutamine, 100 U/mL penicillin and 100 mg/mL streptomycin), typically 5 mL of solution per sample (Additional file 1: Figure S1). Two techniques were done empirically to determine best approach for isolation of MoΦ from atrial tissues. The first technique involved incubation of tissue with collagenase for 30 min while the second technique involved incubation of atrial tissue with collagenase and dispase for 45 min (Additional file 2: Figure S2B) and the second technique was found to be superior and utilized throughout the rest of the study.

In order to activate the digestive enzymes, samples were put on a bench shaker-incubator at 37 °C for 45 min at 250 RPM. Further, samples were rinsed in complete HBSS (HBSS-C, 10% heat-inactivated fetal bovine serum) solution and pushed through a 70 μm filter and washed twice with HBSS-C for 10 min at 400 rcf at 4 °C. Cell isolates were resuspended in FACS buffer (Dulbecco’s phosphate buffered saline, DPBS, 1% bovine serum albumin, 0.1% sodium azide, NaN_3_). Then cell counts and viability were assessed via hemocytometer and trypan blue. Following the counts, cell isolates were purified over a Lymphoprep Ficoll-Paque gradient (GE Healthcare UK, Ltd., Buckinghamshire, UK) and centrifuged for 30 min at 400 rcf with no brake and minimal acceleration. Mononuclear cells at the interphase were collected, counted, and resuspended in FACS buffer and prepared for flow cytometry.

### Blood processing and flow cytometry

**Fig. 1:**
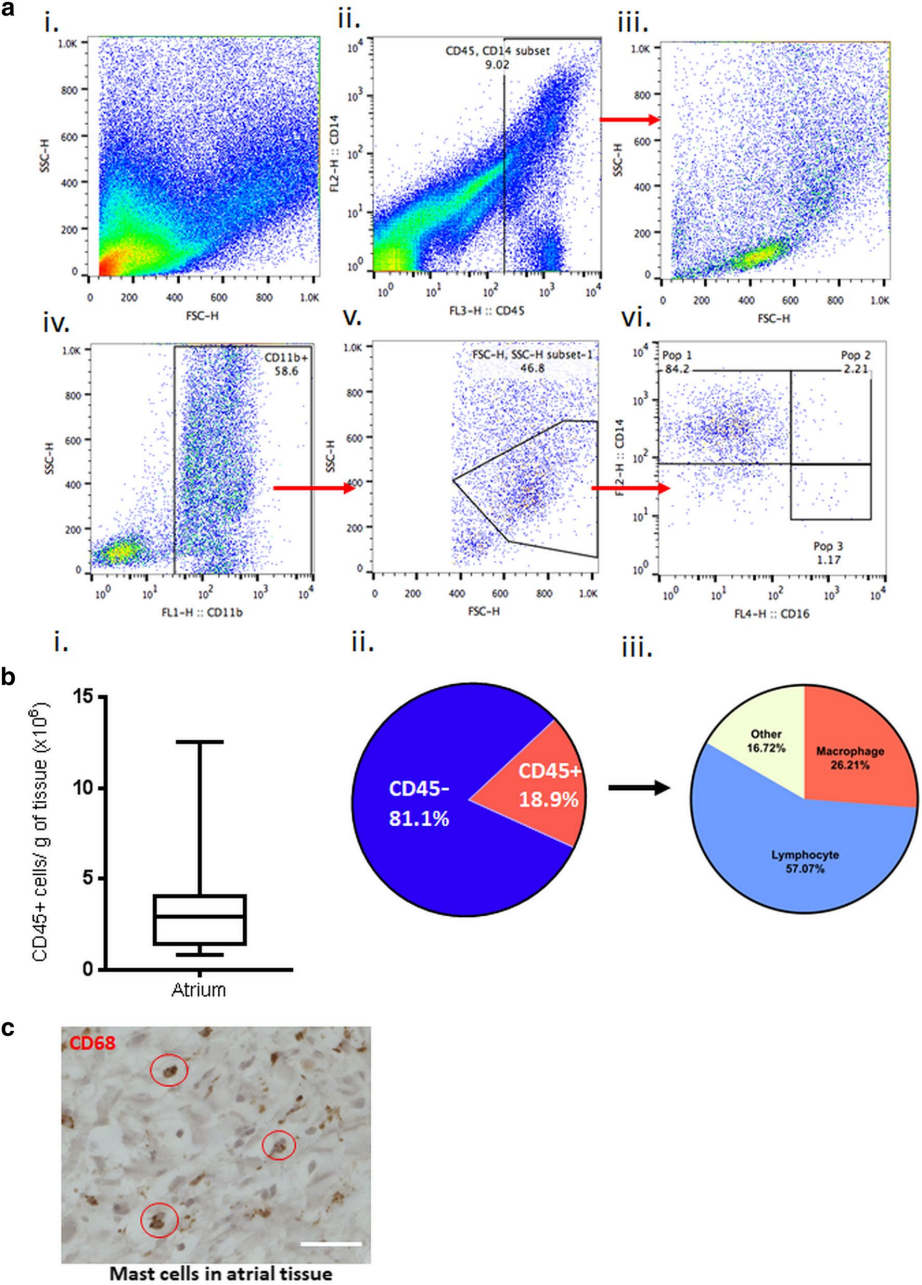
Flow cytometry gating strategy (**a**): (i) Flow cytometry ungated dot plot of overall isolated cells. (ii) CD45 × CD14 gating was applied to exclude non-leukocyte events, resulting in a cleaner population of lymphocytes and MoΦ (iii) Followed by a CD11b x SSC positive gate (iv), then a FSC x SSC gate on MoΦ (v), enabling us to visualize the (vi) different macrophage sub-types with the aid of CD16 × CD14 plot. Leukocytes found in the atrium (**b**): (i) CD45+ cells per gram of tissue calculated from flow cytometry CD45+ data. (ii) Pie chart highlighting the percentage of isolated cells that were CD45+. (iii) Further characterization of the CD45+ population found in atrial samples. **c** Presence of MoΦ in a section of normal human atrial tissue stained with CD68 antibodies. Scale bar represents 400 µm

Heparinized blood samples (100 μL) were incubated with anti-human antibodies linked to different fluorescent labels, CD45-PerCp-Cy5.5 Clone H130 (BioLegend, San Diego, CA), CD11b-FITC Clone ICRF44 (BioLegend, San Diego, CA), CD14-PE Clone 61D3 (eBioscience, San Diego, CA), and CD16-APC Clone eBioCB16 (eBioscience, San Diego, CA). Stained whole blood samples were then incubated with 1× FACS Lysing Solution (BD Biosciences, San Jose, CA) in order to lyse red blood cells. Samples were then fixed with 1% paraformaldehyde solution prepared in PBS with 0.1% sodium azide. Purified atrial mononuclear cell isolations were incubated with the same antibody panel, followed by 1% paraformaldehyde fixation to prepare them for flow cytometry analysis. Isotype controls were used for negative gating to identify the level of non-specific binding. Data were acquired using the FACS Calibur (BD Biosciences, San Jose, CA) then analyzed using FlowJo (Flowjo LLC, Ashland OR). MoΦ gating was performed by open gate on all cells with the use of forward and side scatter (FSC & SSC, Fig. 1ai), and then a gate was applied to all CD45+ cells (wherein there is a CD14+ dominant population, Fig. 1aii). The CD45+ cells were then gated as CD11b+ (Fig. 1aiv) and further focused by FSC & SSC subgating for size and granularity typical of MoΦ (Fig. 1av). The gating on this subgroup of CD11b+ cells were then gated for quantification and displayed for CD16 × CD14 subsets (Fig. 1avi) and to from this CD16+ subset was mean fluorescence intensity (MFI) determined.

### Histological analysis

Atrial heart sections were processed for histological analyses by fixing with 10% neutral buffered formalin for 24 h and then paraffin-embedded. Blocks were sectioned into 5 μm using a microtome and placed on microscope slides for further staining.

### Collagen analysis

In order to assess fibrosis (% collagen deposition), a Sirius Red/Fast Green staining was prepared. Briefly, paraffin-embedded heart sections were stained with Sirius Red then counterstained with Fast Green. Slides were deparaffinized in a series of degrading alcohol gradient and fixed in Bouin’s solution (Sigma) for 1 h. Then, slides were washed in running water until Bouin’s cleared and then placed in 0.1% Fast Green/ddH_2_O for non-collagenous proteins. Slides were then washed in 0.1% acetic acid before staining with 0.1% Sirius red solution for 30 min and then dehydrated and mounted with xylene-based mounting medium. Collagen was semi-quantified using a pixel based method with the use of Adobe Photoshop CC 2017, originally described by Underwood et al. Red pixels were positively selected and summed for a total number of red pixels representing collagen. Nonbackground (green) pixels and red pixels were summed for the total number of heart pixels. Collagen content was measured by calculating the percentage of red pixels by total heart pixels. Consistency was ensured by using the same red colour palette, green colour palette, and by processing all tissues simultaneously.

### Luminex assay and analysis

Approximately 10 mg of human atrial tissue was finely minced and suspended in 1X phosphate buffered saline (PBS) with complete protease inhibitor cocktail (Roche Applied Science, Mannheim, Germany). A Qiagen Tissue Ruptor homogenizer was used to homogenize the tissue. Protein concentrations in the homogenate were determined via Bradford assay (BioRad, Hercules, CA, USA) and the homogenate was stored at − 80 °C. Supernatants collected from human atrial tissue homogenates from patients, were examined using the luminex assay. Fluorescence-coded magnetic micro-particles coated with antibodies specific for the desired cytokines and chemokines were purchased from R&D Systems (Minneapolis, MN, USA) and eBioscience (San Diego, CA, USA). The luminex assay was conducted according to manufacturer’s instructions. The samples were read using a Bio-Rad Bio-Plex dual laser (BioRad, Hercules, CA, USA). A reference value of normal atrial tissue was used as a qualifier.

### Data analysis

All data analysis was performed using GraphPad Prism 6 (GraphPad Software Inc, La Jolla, CA). Categorical variables were reported as frequencies and percentages and were analyzed by chi-square. Dropouts in grouping values for luminex (i.e. less than expected, N = 9 SR v. N = 9 AFib), were either: high-range outliers identified by the ROUT method with a Q = 0.1% specificity or samples that failed to be assayed (ex. insufficient surgical sample available, clotting or interference due to hemolysis). Individual samples were not included in a group average if the sample was determined to be lower than the LLD of the assay and in that instance the LLD were indicated on the bar graph. Unpaired two-tailed t-tests were carried out to compare two groups, or otherwise two-tailed Mann–Whitney non-parametric test; with P < 0.5 was considered significantly different.

## Results

### Patient characteristics

Atrial tissue samples were obtained for analysis in 18 patients. Patient characteristics are outlined in Table 1. The majority of patients were males (78%), with normal ejection fraction (72%), required surgery while in hospital (56%), and undergoing various cardiac surgical interventions ranging from CABG, valve, combined procedures and LVAD implantation for chronic heart failure. All patients received standard of care at a tertiary cardiac surgery center performing nearly 1000 cardiac surgical interventions per year [21].

**Table 1:** Baseline characteristics of patients cohort

Parameters	SR (n = 9)	AF (n = 9)	Patients (n = 18)	P-value
Age	62 ± 3.3	67 ± 3.0	64.1 (44–84)	0.229
Female gender	22	22	22%	1.000
Diabetes	11	11	11%	1.000
EF	59 ± 4.9	44 ± 7.2	51.3 (16–78)	0.114
EF > 50%	8	5	44%	0.200
BMI	27.6 ± 1.9	31.7 ± 3.2	29.6 (20–53.7)	0.292
NYHA III	1	5	66%	0.049*
Urgency				
In-hospital	4	5	50%	0.637
Elective	5	4	50%	
Procedure type				
CABG	4	4	8/18	0.940
CABG + valve	1	1	2/18	
Aortic valve	3	1	4/18	
Aortic + mitral valve	1	1	2/18	
LVAD	1	1	2/18	
Clinical frailty				
CFS class 4/5	3	6	50%	0.157
Echocardiogram				
LV diast diameter	5.1 ± 0.6	5.7 ± 1.1	5.5 (4.0–7.3)	0.422
LV syst diameter	3.5 ± 0.9	4.3 ± 1.9	4.0 (2.4–6.9)	0.623
LA diameter	4.2 ± 0.8	11.1 ± 16.7	4.5 (2.8–4.9)	0.295
E/A ratio	1.4 ± 1.2	0.9 ± 0.1	1.2 (0.5–3.7)	0.845
CPB				
Pump time	120 ± 38.3	107.5 ± 33.1	114.1 (65–187)	0.689
LOS (days)	9 ± 1.9	17 ± 3.1	12.8 (5–27)	0.026*
Adverse events				
Delirium	2	3	5/18	0.598
Stroke/TIA	0	1	1/18	1.000
CHF	2	5	7/18	0.050*

*EF* ejection fraction, *CABG* coronary artery bypass graft, *BMI* body mass index, *CPB* cardiopulmonary bypass)

*Statistically significant

### Atrial tissue is composed largely of CD45+ cells

The mean size of the piece of right atrium collected was 0.41 ± 0.06 g (Additional file 2: Figure S2). A standardized approach to tissue processing was carried out which allowed the isolation and purification of 3.21 × 10^6^ ± 0.66 PBMC/g of tissue. Flow cytometry gating strategy was standardized so that all samples were analyzed with a similar approach and strategy to allow comparison (Fig. 1a). Leukocytes were identified with flow cytometry from the isolates using CD45+ positivity. The recovery rate of the PBMC isolation used was 18.91 ± 2.33% with a total of 0.66 × 10^6^ ± 0.25 CD45+ cells per gram of tissue were found in purified isolates from the atrium representing 18.9% of the total cells isolated per gram of atrial tissue (Fig. 1b(i, ii)). Using SSC and FSC distribution after CD45 gating we were able to determine that the majority of PBMCs were lymphocytes (57%) compared to MoΦ (26%) with 17% other or unclear (Fig. 1b(iii)). MoΦ showed a statistically significant difference in CD45 MFI in AFib patients compared to those in S.R. (Fig. 3d). Similarly, the CD45 MFI for lymphocytes was also higher in AFib patients with P = 0.05. Note that gating on lymphocytes allowed the identification a significant population of CD16+ lymphocytes suggestive of a population of NK cells.

**Fig. 2:**
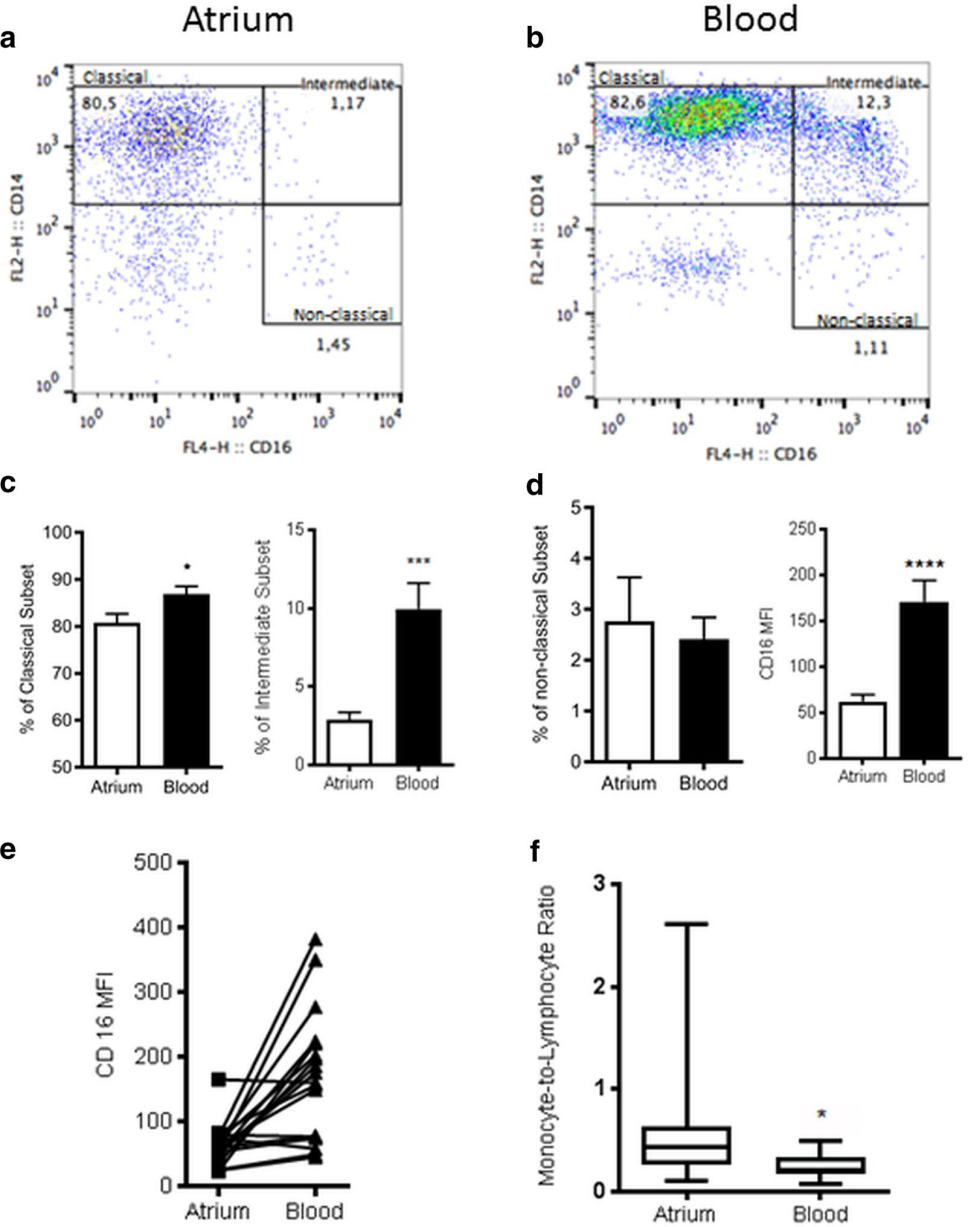
**a**, **b** Flow cytometry dot plots from an individual patient’s atrium and blood respectively. **c** Bar graphs demonstrating the comparison between the different MoΦ phenotypes in atrium and blood. **d** CD16 MFI values in atrium and blood. **e** Individual patient data on CD16 MFI to further show that CD16 MFI shows a similar trend in all patients where CD16 MFI is lower in atrium than in blood. **f** MoΦ to lymphocyte ratio was significantly higher in atrial samples

The monocytes/macrophages (MoΦ) distribution was then further characterized based on CD14 and CD16 expression. The vast majority of MoΦ appeared to be classical CD14++/CD16− (81%) with fewer CD14++/CD16+ intermediate (3%) and CD14+/CD16+ non-classical (1%) (Fig. 2a, Additional file 3: Figure S3). Comparisons were made between PBMC findings from atrial isolates and peripheral blood to ensure that findings from atrial isolation would not be reflective of blood contamination. Findings from blood samples were significantly different from atrial isolates. Specifically, CD16 MFI was significantly higher in peripheral blood MoΦ in keeping with greater proportion intermediate and non-classical MoΦ when compared to cells seen in tissue (Fig. 2b, d). Findings from individual patients can be seen in Fig. 2e illustrating that in the majority of patients CD16 MFI was significantly different between atrial isolates and peripheral blood.

### Histological analysis of atrial fibrosis showed strong correlation with age but did not exhibit any significant difference between AFib and SR patients

**Fig. 3:**
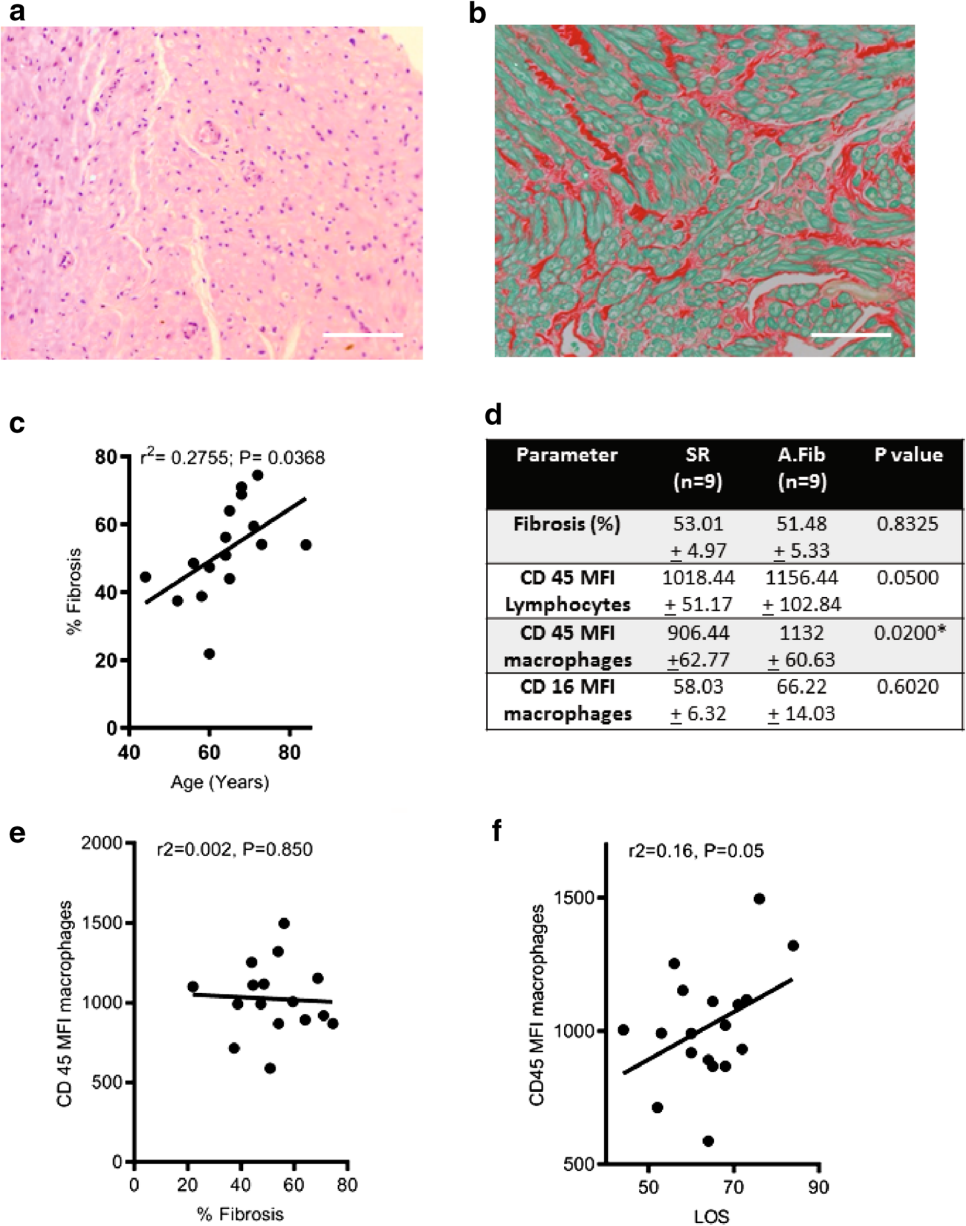
**a** H&E stain of normal human atrium. Scale bar represents 200 µm. **b** Sirius red stain showing collagen fibers stained red, and myocardial fibers stained green. Scale bar represents 100 µm. **c** graph showing fibrosis was positively correlated with patient age. **d** table representing salient differences between patients in SR and AFib. **e** CD45 MFI MoΦ shows no correlation to percent fibrosis. **f** CD45 MoΦ show statistically significant correlation to hospital length of stay

All samples were processed and stained for routine histological analysis using H&E and Sirius Red staining for collagen deposition. Histological analysis was performed by a single anatomical pathologist blinded to the study patients. A representative image of atrial tissue is seen (Fig. 3a) demonstrating that by routine H&E no apparent myocardial inflammatory infiltrates could be identified. Immunohistochemistry was used to identify the presence of leukocytes. CD68 was used to identify MoΦ and cells were quantified in tissue sections (Fig. 1c). In the representative image CD68+ cells can be identified within the atrial myocardium confirming the presence of immune cells within the atrial tissue as demonstrated from flow cytometry findings.

Significant tissue collagen was seen on routine H&E and Sirius Red stained slides with mild or more fibrosis (semi-quantitative interpretation) seen in 87% of patients (Fig. 3b). Quantification of fibrosis was performed from histological specimens using a well-described approach using Sirius Red stain. Fibrosis was positively correlated with patient age (r^2^ = 0.28; P = 0.037) (Fig. 3c). Interestingly, there was no significant difference in fibrosis when patients in SR were compared with those in AFib (Fig. 3d).

### AFib patients show higher expression of MMP-9 in atrial tissue

**Fig. 4:**
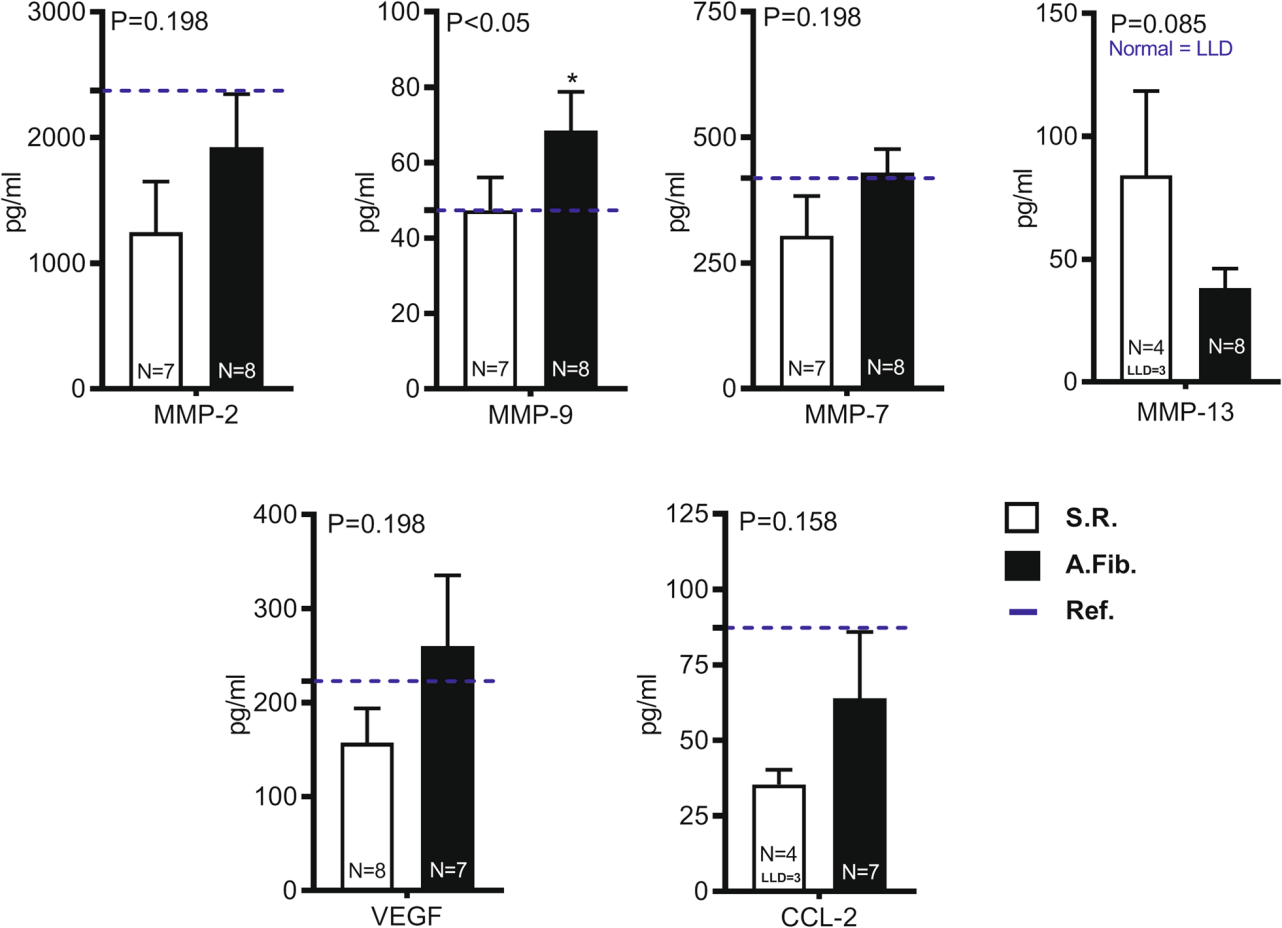
Luminex analyses of atrial tissue isolates comparing SR and AFib patients with a normal atrial tissue used as reference value. MMP-9 was statistically significant in AFib patients with P < 0.05. Other cytokines highly expressed in AFib patients were MMP-2, MMP-7, VEGF and CCL-2/MCP-1

**Fig. 5:**
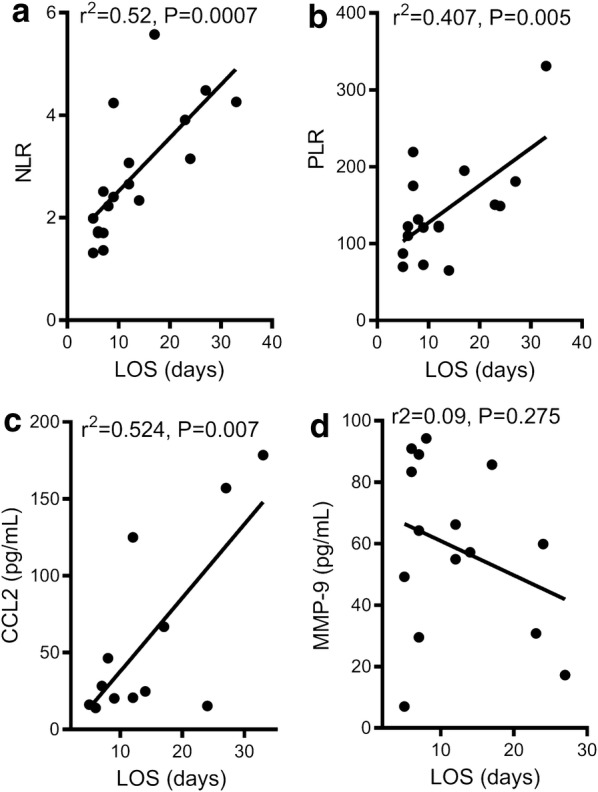
Neutrophil to Lymphocyte ratio (NLR), Platelet to lymphocyte ration (PLR), and CCL-2/MCP-1 were positively correlated with hospital length of stay (LOS). MMP-9 a diagnostic indicator was negatively correlated to LOS

A luminex panel was used to quantify the expression of proteins previously suggested to be important in fibrogenesis. Patients were grouped by the diagnosis of AFib and compared to patients in SR. All findings were compared to normal atrial tissue obtained from a donor heart tissue (historical sample taken at the time of harvest from a single donor), which served as a control (reference value). There was higher expression of pro-inflammatory cytokines like VEGF and CCL-2/MCP-1 in patients with AFib (Fig. 4). Metalloproteinases that were highly expressed in AFib patients were MMP-2, MMP-7, with MMP-9 being statistically significant and highly expressed in AFib patients. The other cytokines that were included in the panel but were below the lower limit of detection (LLD) were TNF, GM-CSF, CCL-7, interleukins (ILs)-1B, 4, 5, 8, 10, 13, 17A, and 33. Additional cytokines that were significantly elevated in AFib patients were GDF-15, TNFR2, NT-propBNP (Additional file 4: Figure S4). Though not discriminating AFib, CCL-2/MCP-1 was found to positively correlate with hospital length of stay (Fig. 5)

### AFib patients had more comorbidity, longer stay in hospital that correlated with positively with MFI-CD45+ cells density in the atria

There was no in-hospital mortality and the median length of hospitalization was 9 days (IQR 7–19). Fibrosis appeared to positively correlate with age (Fig. 3c). If patients were grouped based on age (arbitrarily 65) fibrosis was significantly higher in patients > 65 (45.4 ± 3.6 vs 63.7 ± 3.7; P = 0.0049) and CD45+ MFI for MoΦ was also significantly higher (928.8 ± 51.9 vs 1161.3 ± 77.1; P = 0.019) (Fig. 3d). AFib was the primary outcome of interest. One third of patients had pre-operative AFib, but in total 50% of patients (including post-op) had AFib documented during the indexed hospitalization. Using this approach we were not able to demonstrate that AFib was significantly different between the two groups. However, we were able to demonstrate that CD45+ MFI was significantly higher in patients with AFib suggesting higher number of MoΦ. There were significant differences in CD45 MFI of MoΦ and lymphocytes in AFib patients compared to those in SR, while there was no significant difference in CD16 MFI of MoΦ in AFib patients (Fig. 3d). CD45 MFI of MoΦ showed no correlation to fibrosis (Fig. 3e), however, CD45 MFI of MoΦ was correlated significantly to hospital length of stay (Fig. 3f).

Patients suffering from AFib were more frail, had a higher BMI, higher NYHA classification and had lower ejection fractions. Additionally, AFib patients also had higher incidences of adverse events like chronic heart failure and longer lengths of stay in hospital. Similar to what was seen with CCL2 and MMP-9 additional markers of inflammation, namely NLR and PLR, were positively correlated to hospital length of stay (Fig. 5).

## Discussion

To our knowledge, we are the first to successfully isolate mononuclear cells from the human atrium for FACS analysis in order to identify linkages with clinical information. Here we show a combination of defined cellular and cytokine quantification that could be used to inform clinical decision making. We also corroborate further findings that indicate fibrosis is correlated with age but does not segregate AFib patients from patients with normal SR. Rather, we find that AFib patients are encumbered by leukocyte infiltration and inflammatory biomarkers in a manner that correlates well with surrogates of overall outcome, including length of stay. This information can now provide a framework by which key biomarkers, like MMPs, could provide potential diagnostic indicators of AFib, while chemotactic proteins could be prognostic of clinical outcomes. Utilizing this combined approach of cell-cytokine analysis of tissue derived from cardiac surgical standard care could lead to the development of panels to help guide diagnostic and therapeutic strategies.

Fibrosis is broadly considered to be the cornerstone of AFib pathology [14, 15] and that structural remodeling and fibrotic replacement of the atrium is causative to onset and severity of AFib. Our findings are controversial in this regard, as we found no difference in fibrosis that was not coincidently an age affect between AFib and SR patients, which others have reported previously [18]. The classical view of atrial structural remodeling or fibrosis linked to disturbances in ECM turnover is caused by TGFβ1 and AngII levels higher in patients at risk of post-op AFib [22]. Others have also questioned the causal link of AFib to fibrosis. In a series of 46 patients undergoing cardiac surgery, the right and left atria were sampled and no difference in ECM content could be demonstrated between AFib and SR groups [23]. It is important to consider fibrosis as independent to the drive of AFib but this doesn’t necessarily mean it is inconsequential. We showed high collagen deposition in the atria of aging patients and this would be expected to slow atrial conduction and increase susceptibility or exacerbate AFib [17, 24], but may not be a driver per se. Though we also show that older patients had increased numbers of CD45+ MoΦ in the atrium, it was only in the AFib population that a higher CD45+ MoΦ reached showed significance compared to SR patients. It has also been previously shown that structural remodeling of the atria occurs independent of cardiovascular comorbidities and the presence of AFib [25]. Further, previous findings have shown atrial remodeling occurs in non-cardiac conditions like cancer, sepsis, kidney disease without developing into AFib [26]. Taken together we should begin to question whether fibrosis is contributing to the incipient cause of AFib or is more likely a co-existing product due to other comorbidities or age.

In the context of atrial tissue, inflammation is associated with atrial remodeling and the substrate by which AFib develops [27, 28]. Most of the evidence describing resident MoΦ has been obtained from mice models and rarely has human tissue evidence been a focus. There has been a prevailing dogma that immune cells in heart tissue were mainly derived from the circulation, which led investigators to largely disregard resident immune cells. Most studies have looked at the atrium for signs of inflammation [23, 29], however this approach has been proven to be limited and largely unable to characterize resident immune cells (MoΦ) that are increasingly recognized as an important component of heart tissue [12, 30, 31]. Also, MoΦ in particular have been shown to be capable of influencing the complex balance between inflammation and its’ resolution [32, 33]. MoΦ appear particularly important given their ability to display a large array of activation states ranging from pro-inflammatory (classical) to immunomodulatory (non-classical) [32, 33]. Initial work by Pinto et al. suggested that resident MoΦ were largely an non-classical-like phenotype or alternatively activated [34–36]. We demonstrate that a large population of MoΦ isolated from human atrium are pro-inflammatory CD14++/CD16− (classical) with only a small proportion of CD16+ MoΦ (non-classical). Our approach to the isolation was rigorous, standardized and adapted from identical methodology previously described by our group [12, 13]. Our findings may reflect inherent differences between human and mice, most notably: mice do not express CD16 but instead Ly6c, a marker that has been necessary to identify polarity in mice [37]. Furthermore, recent evidence indicates that embryonic resident MoΦ are gradually replaced with MoΦ from the circulation, significantly reducing the non-classical phenotype by adulthood as we have observed in our population favoring the classical phenotype [38]. We demonstrated that the isolated MoΦ phenotype from atrial cells was different than circulating MoΦ suggesting that our isolation strategy did not result simply from blood contamination. Our isolation strategy was designed to characterize MoΦ, while eliminating neutrophils. Neutrophils were not seen on routine histology sections and as such, do not likely represent on large number of cells. On the other hand, a large population of lymphocytes was identified based on CD45+, SSC and FSC distribution. Our finding of pro-inflammatory macrophage infiltration in the atrium corroborates previous work showing increased MoΦ accumulation in the atria of AFib patients [39, 40]. Additional markers were not used to further characterize these lymphocytes in part by the limitations imposed by the size of tissue sampled and the number of cells obtained. However, it is noteworthy that a significant population of lymphocytes appears to be CD16+ suggesting that a population of NK cells can be isolated from the atrium [41].

**Fig. 6:**
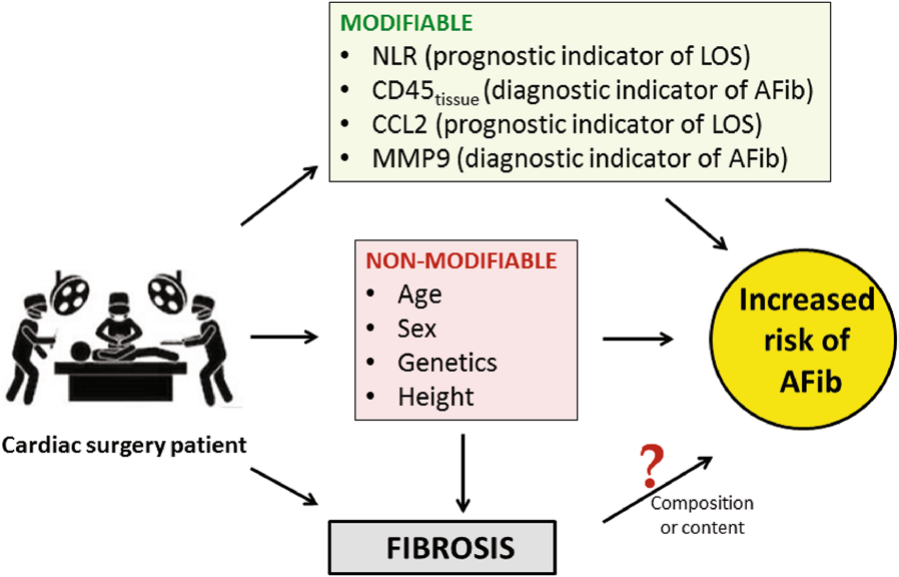
Clinical overview of a potential algorithm to the modifiable and non-modifiable factors of the cardiac surgery patient’s risk for AFib. The modifiable factors are inflammatory, some having prognostic value determined through a LOS surrogate, such as NLR and CCL2, and some with diagnostic value determined as the onset of AFib, such as CD45 and MMP9. Utility of these biomarkers would be independent of on-modifiable factors such as age, sex, height and genetics, which can also contribute to the development of fibrosis in AFib patients. However, fibrosis is to be questioned as to whether it is an incipient cause or correlative pre-condition to AFib in patients, experimental models should be developed to answer this question definitively

In addition to defining cellular content, the inflammatory milieu from those atrial MoΦ should present the opportunity specific biomarker identification which when used together could provide a panel that informs diagnostic/therapeutic decision making (Fig. 6). MMPs are extremely potent protein degradation and modifying enzymes and are key factors in ECM remodeling of the atrium thereby propagating the development of AFib. Our results showed higher expression of MMP-2, 7 and 9, the key gelatinases involved in ECM degradation in AFib patients corroborating previous findings on human samples [42, 43], while elevated MMP-7 was seen in rats with mild and severe heart failure. This suggests that there is atrial structural remodeling associated with the development of sustained AFib in these patients. Previous studies have shown that patients with higher serum levels of MMP-2 were at a higher risk of failure to restore SR [44], suggesting its potential role as a prognostic biomarker to stratify patients pre-operatively for better management of AFib. Also, previous studies have shown that circulating levels of MMPs in combination could increase the prediction of AFib and development of biomarker panels for prognosis and patient stratification. VEGF is another pro-inflammatory cytokine has been implicated in AFib [45], and was found to be elevated in AFib patients along with MMP-9 as we have shown [46]. The other cytokines that were not detected in our experiments have been shown to be inconsistently measured in other trials as well [45], and may not be useful as prognostic tools in AFib patients. Interestingly, CCL2 was positively correlated with hospital length of stay reaffirming its role as a potential prognostic marker AFib (Fig. 6). Previous studies have shown increased expression of CCL2 genes in the murine atrium [47] which regulates chemotaxis and myeloperoxidase production in the left atrium, linked to the development of AFib and atrial fibrosis [42]. CCL2 is also responsible for macrophage recruitment in injured tissues suggesting its crucial role in acute inflammation, repair, fibrosis and chronic inflammation. We propose a clinical algorithm that could be the focus of future mechanistic studies. MMP-9 could potentially serve as a diagnostic indicator of AFib and CCL-2/MCP-1 could be a prognostic biomarker of AFib potentially in combination with NLR, that are also predictors of clinical outcomes like hospital length of stay (Fig. 6).

Limitations of the present study are the small sample size and differences between patients and groups cannot be fully accounted for. Though this is a small study it is the first to combine cellular and cytokine information to reconcile with clinical findings related to Afib. In so doing, we contribute to a growing body of evidence that suggests AFib substrate is not contingent on the degree of fibrosis. However, we cannot at this time rule out fibrotic composition (i.e. quantity vs quality of ECM remodeling). For the same reasons it was not possible to create multivariable models with only 18 patients. Future studies are now warranted to expand and determine with greater confidence the diagnostic and prognostic value of atrial cellular-cytokine analyses related to outcomes. Differences in methodologies, patient populations, type of Afib, presence of left ventricular dysfunction, age, gender, renal clearance and HTN all contribute to study results, and couldn’t be controlled for in the present study [28]. Our study also illustrates that while cell isolation is possible from atrial tissue it does represent significant technical challenges including but not limited to cell loss that could mean that our findings are not reflective of the whole heart. The luminex measurements were a single point of time measures that are not reflective of the dynamic inflammatory environment.

Macrophage classification into M1 and M2 phenotypes were limited to CD14 and CD16 expression in this study. Additional M1 markers that could be used are CD80, CD68, iNOS, and M2 markers like CD163, CD206 would help classify MoΦ further. However, the concept of M1/M2 classification of MoΦ is broad and overinterpreted. Whether MoΦ are themselves disease causing, and therefore disease-specific or simply associated with disease in unique ways remains to be fully determined. Given the limited access to normal atrial tissue, only one control was used, permitting guarded interpretations of study results. Finally, one needs to acknowledge that our atrial sampling was mainly of the right atrial appendage, which may not reflect findings from the entire atrium or findings from the left atrium, which were not assessed in the present study.

## Conclusions

In the present study we demonstrate that a significant population of inflammatory cells can be isolated from the atrium of patients undergoing heart surgery. Our findings also suggest that more inflammatory cells can be seen in patients at risk of post-operative AFib supporting a mechanistic link between inflammation and AFib, but not necessarily fibrosis. Biomarkers associated with inflammation, particularly oxidative stress biomarkers were shown to be associated with post-op AFib [8]. Our data showed higher expression of inflammatory cytokines like VEGF and CCL2 with ~ 1.5-fold increases in MMPs 2, 7, and 9 in AFib patients.

## Supplementary information

**Additional file 1: Figure S1.** (1) The right atrial appendage is finely minced and (2) treated with collagenase and dispase in serum free HBBS media. (3) The cells are then filtered through a 70um filter and washed twice to isolate cells. (4) Cells are isolated over Lymphoprep and centrifuged for 30 min at 400G with no brake. (5) Mononuclear cells at the interphase are isolated and (6) incubated with anti-human antibodies linked to different fluorescent labels for (7) flow cytometry analysis.

**Additional file 2: Figure S2.** Macrophage isolation purity. (A) Dot plot representing the variability of obtainable tissue weight. (B) PBMC isolation comparison between two different techniques. Technique 1: incubation of tissue with collagenase for 30 min. Technique 2: incubation of tissue with collagenase + dispase for 45 min. Technique 2 yielded a higher cell isolation that was significantly different from technique 1 (P = 0.02).

**Additional file 3: Figure S3.** Flow cytometry macrophage subset analysis. (A) Flow cytometry dot plot representing gated macrophage populations (CD16 x CD14) as a mean value (n = 18). (B) CD16 MFI values in atrial tissue isolates.

**Additional file 4: Figure S4.** Luminex analyses of cytokines in AFib, SR and control patients. Cytokines that were significantly elevated in AFib patients were GDF-15, TNFR2, NT-propBNP.

## Data Availability

The datasets used and/or analysed during the current study are available from the corresponding author on reasonable request.

## Abbreviations

PSR: picrosirius red
CD: cluster of differentiation
AFib: atrial fibrillation
SR: sinus rhythm
CCL-2/MCP-1: chemokine ligand 2 or monocyte chemoattractant protein 1
MMP-9: matrix metalloproteinase 9
NLR: neutrophil to lymphocyte ratio
ECM: extra cellular matrix
TNF-α: tumor necrosis factor α
CRP: C-reactive protein
IL: interleukin
VEGF: vascular endothelial growth factor
GM-CSF: granulocyte-macrophage colony-stimulating factor
RNA: ribonucleic acid
ACT: activated clotting time
CABG: coronary artery bypass grafting
NYHA: New York heart association
LOS: length of stay
HBSS: Hank’s balanced salt solution
RPM: rotations per minute
FACS: fluorescence-activated cell sorter
DPBS: Dulbecco’s phosphate buffered saline
LLD: lower limit of detection
LVAD: left ventricular assist device
PBMC: peripheral blood mononuclear cells
MFI: mean fluorescence intensity
H&E: Hematoxylin and Eosin
RAAS: renin–angiotensin–aldosterone system
SSC: side-scattered light
FSC: forward scattered light
NK: natural killer cells
HTN: hypertension

## References

[1] Heart and Stroke. Atrial fibrillation; 2018. http://www.heartandstroke.ca/heart/conditions/atrial-fibrillation. Accessed 15 Oct 2018.

[2] Centers for Disease Control and Prevention (CDC); 2017. https://www.cdc.gov/dhdsp/data_statistics/fact_sheets/fs_atrial_fibrillation.htm. Accessed 22 Aug 2017.

[3] Rienstra M, Lubitz SA, Mahida S, Magnani JW, Fontes JD, Sinner MF (2012). Symptoms and functional status of patients with atrial fibrillation: state of the art and future research opportunities. Circulation..

[4] Norby FL, Soliman EZ, Chen LY, Bengtson LG, Loehr LR, Agarwal SK (2016). Trajectories of cardiovascular risk factors and incidence of atrial fibrillation over a 25-year follow-up: the ARIC Study (Atherosclerosis Risk in Communities). Circulation..

[5] Dzeshka MS, Lip GYH, Snezhitskiy V, Shantsila E (2015). Cardiac fibrosis in patients with Atrial Fibrillation. Mech Clin Implic.

[6] Andrade J, Khairy P, Dobrev D, Nattel S (2014). The clinical profile and pathophysiology of atrial fibrillation: relationships among clinical features, epidemiology, and mechanisms. Circ Res.

[7] Hu YF, Chen YJ, Lin YJ, Chen SA (2015). Inflammation and the pathogenesis of atrial fibrillation. Nat Rev Cardiol.

[8] Wu JH, Marchioli R, Silletta MG, Masson S, Sellke FW, Libby P (2015). Oxidative stress biomarkers and incidence of postoperative atrial fibrillation in the omega-3 fatty acids for prevention of postoperative atrial fibrillation (OPERA) trial. J Am Heart Assoc..

[9] Harada M, Van Wagoner DR, Nattel S (2015). Role of inflammation in atrial fibrillation pathophysiology and management. Circ J.

[10] Gawdat K, Legere S, Wong C, Myers T, Marshall JS, Hassan A (2017). Changes in Circulating Monocyte Subsets (CD16 Expression) and Neutrophil-to-Lymphocyte Ratio Observed in Patients Undergoing Cardiac Surgery. Front Cardiovasc Med.

[11] Shao Q, Chen K, Rha SW, Lim HE, Li G, Liu T (2015). Usefulness of neutrophil/lymphocyte ratio as a predictor of atrial fibrillation: a meta-analysis. Arch Med Res.

[12] Falkenham A, Myers T, Wong C, Legare JF (2016). Implications for the role of macrophages in a model of myocardial fibrosis: CCR2(-/-) mice exhibit an M2 phenotypic shift in resident cardiac macrophages. Cardiovasc Pathol..

[13] Falkenham A, de Antueno R, Rosin N, Betsch D, Lee TD, Duncan R (2015). Nonclassical resident macrophages are important determinants in the development of myocardial fibrosis. Am J Pathol.

[14] Yamashita T, Sekiguchi A, Iwasaki YK, Date T, Sagara K, Tanabe H (2010). Recruitment of immune cells across atrial endocardium in human atrial fibrillation. Circ J.

[15] Hu YF, Chen YJ, Lin YJ, Chen SA (2015). Inflammation and the pathogenesis of atrial fibrillation. Nat Rev Cardiol.

[16] Corradi D (2014). Atrial fibrillation from the pathologist’s perspective. Cardiovasc Pathol..

[17] Dzeshka MS, Lip GY, Snezhitskiy V, Shantsila E (2015). Cardiac fibrosis in patients with atrial fibrillation: mechanisms and clinical implications. J Am Coll Cardiol..

[18] Cochet H, Mouries A, Nivet H, Sacher F, Derval N, Denis A (2015). Age, atrial fibrillation, and structural heart disease are the main determinants of left atrial fibrosis detected by delayed-enhanced magnetic resonance imaging in a general cardiology population. J Cardiovasc Electrophysiol.

[19] Rosin NL, Sopel MJ, Falkenham A, Lee TD, Legare JF (2015). Disruption of collagen homeostasis can reverse established age-related myocardial fibrosis. Am J Pathol.

[20] Fernandez FG, Falcoz PE, Kozower BD, Salati M, Wright CD, Brunelli A (2015). The Society of Thoracic Surgeons and the European Society of Thoracic Surgeons general thoracic surgery databases: joint standardization of variable definitions and terminology. Ann Thorac Surg.

[21] Buth KJ, Gainer RA, Legare JF, Hirsch GM (2014). The changing face of cardiac surgery: practice patterns and outcomes 2001-2010. Can J Cardiol.

[22] Swartz MF, Fink GW, Sarwar MF, Hicks GL, Yu Y, Hu R (2012). Elevated pre-operative serum peptides for collagen I and III synthesis result in post-surgical atrial fibrillation. J Am Coll Cardiol.

[23] Smorodinova N, Lantova L, Blaha M, Melenovsky V, Hanzelka J, Pirk J (2015). Bioptic study of left and right atrial interstitium in cardiac patients with and without atrial fibrillation: interatrial but not rhythm-based differences. PLoS ONE.

[24] Jansen HJ, Moghtadaei M, Mackasey M, Rafferty SA, Bogachev O, Sapp JL (2017). Atrial structure, function and arrhythmogenesis in aged and frail mice. Scientific Rep.

[25] Platonov PG (2017). Atrial fibrosis: an obligatory component of arrhythmia mechanisms in atrial fibrillation?. J Geriatr Cardiol JGC..

[26] Ferreira C, Providência R, Ferreira MJ, Gonçalves LM (2015). Atrial fibrillation and non-cardiovascular diseases: a systematic review. Arq Bras Cardiol.

[27] Patel P, Dokainish H, Tsai P, Lakkis N (2010). Update on the association of inflammation and atrial fibrillation. J Cardiovasc Electrophysiol.

[28] Turagam MK, Mirza M, Werner PH, Sra J, Kress DC, Tajik AJ (2016). Circulating biomarkers predictive of postoperative atrial fibrillation. Cardiol Rev..

[29] Mitrofanova LB, Orshanskaya V, Ho SY, Platonov PG (2016). Histological evidence of inflammatory reaction associated with fibrosis in the atrial and ventricular walls in a case-control study of patients with history of atrial fibrillation. Europace..

[30] Pinto AR, Ilinykh A, Ivey MJ, Kuwabara JT, D’Antoni ML, Debuque R (2016). Revisiting Cardiac Cellular Composition. Circ Res.

[31] Li SH, Sun Z, Brunt KR, Shi X, Chen MS, Weisel RD (2013). Reconstitution of aged bone marrow with young cells repopulates cardiac-resident bone marrow-derived progenitor cells and prevents cardiac dysfunction after a myocardial infarction. European heart journal..

[32] Murray PJ, Wynn TA (2011). Protective and pathogenic functions of macrophage subsets. Nat Rev Immunol.

[33] Duffield JS, Lupher M, Thannickal VJ, Wynn TA (2013). Host responses in tissue repair and fibrosis. Annu Rev Pathol.

[34] Epelman S, Lavine KJ, Beaudin AE, Sojka DK, Carrero JA, Calderon B (2014). Embryonic and adult-derived resident cardiac macrophages are maintained through distinct mechanisms at steady state and during inflammation. Immunity.

[35] Heidt T, Courties G, Dutta P, Sager HB, Sebas M, Iwamoto Y (2014). Differential contribution of monocytes to heart macrophages in steady-state and after myocardial infarction. Circ Res.

[36] Pinto AR, Paolicelli R, Salimova E, Gospocic J, Slonimsky E, Bilbao-Cortes D (2012). An abundant tissue macrophage population in the adult murine heart with a distinct alternatively-activated macrophage profile. PLoS ONE.

[37] Yang J, Zhang L, Yu C, Yang X-F, Wang H (2014). Monocyte and macrophage differentiation: circulation inflammatory monocyte as biomarker for inflammatory diseases. Biomark Res.

[38] Lavine KJ, Epelman S, Uchida K, Weber KJ, Nichols CG, Schilling JD (2014). Distinct macrophage lineages contribute to disparate patterns of cardiac recovery and remodeling in the neonatal and adult heart. Proc Natl Acad Sci USA.

[39] Sun Z, Zhou D, Xie X, Wang S, Wang Z, Zhao W (2016). Cross-talk between macrophages and atrial myocytes in atrial fibrillation. Basic Res Cardiol.

[40] He G, Tan W, Wang B, Chen J, Li G, Zhu S (2016). Increased M1 macrophages infiltration is associated with thrombogenesis in rheumatic mitral stenosis patients with atrial fibrillation. PLoS ONE..

[41] Uciechowski P, Werfel T, Leo R, Gessner JE, Schubert J, Schmidt RE (1992). Analysis of CD16+ dim and CD16+ bright lymphocytes–comparison of peripheral and clonal non-MHC-restricted T cells and NK cells. Immunobiology..

[42] Rudolph V, Andrie RP, Rudolph TK, Friedrichs K, Klinke A, Hirsch-Hoffmann B (2010). Myeloperoxidase acts as a profibrotic mediator of atrial fibrillation. Nat Med.

[43] Nakano Y, Niida S, Dote K, Takenaka S, Hirao H, Miura F (2004). Matrix metalloproteinase-9 contributes to human atrial remodeling during atrial fibrillation. J Am Coll Cardiol..

[44] Kato K, Fujimaki T, Yoshida T, Oguri M, Yajima K, Hibino T (2009). Impact of matrix metalloproteinase-2 levels on long-term outcome following pharmacological or electrical cardioversion in patients with atrial fibrillation. Europace.

[45] Scridon A, Morel E, Nonin-Babary E, Girerd N, Fernandez C, Chevalier P (2012). Increased intracardiac vascular endothelial growth factor levels in patients with paroxysmal, but not persistent atrial fibrillation. EP Europace..

[46] Ogi H, Nakano Y, Niida S, Dote K, Hirai Y, Suenari K (2010). Is structural remodeling of fibrillated atria the consequence of tissue hypoxia?. Circ J.

[47] Kahr PC, Piccini I, Fabritz L, Greber B, Scholer H, Scheld HH (2011). Systematic analysis of gene expression differences between left and right atria in different mouse strains and in human atrial tissue. PLoS ONE.

