# Supplementary figures and images for: Fibrosis independent atrial fibrillation in older patients is driven by substrate leukocyte infiltration: diagnostic and prognostic implications to patients undergoing cardiac surgery
Source: J Transl Med. 2019 Dec 10;17:413. doi: 10.1186/s12967-019-02162-5 (PMC6905054; doi:10.1186/s12967-019-02162-5)

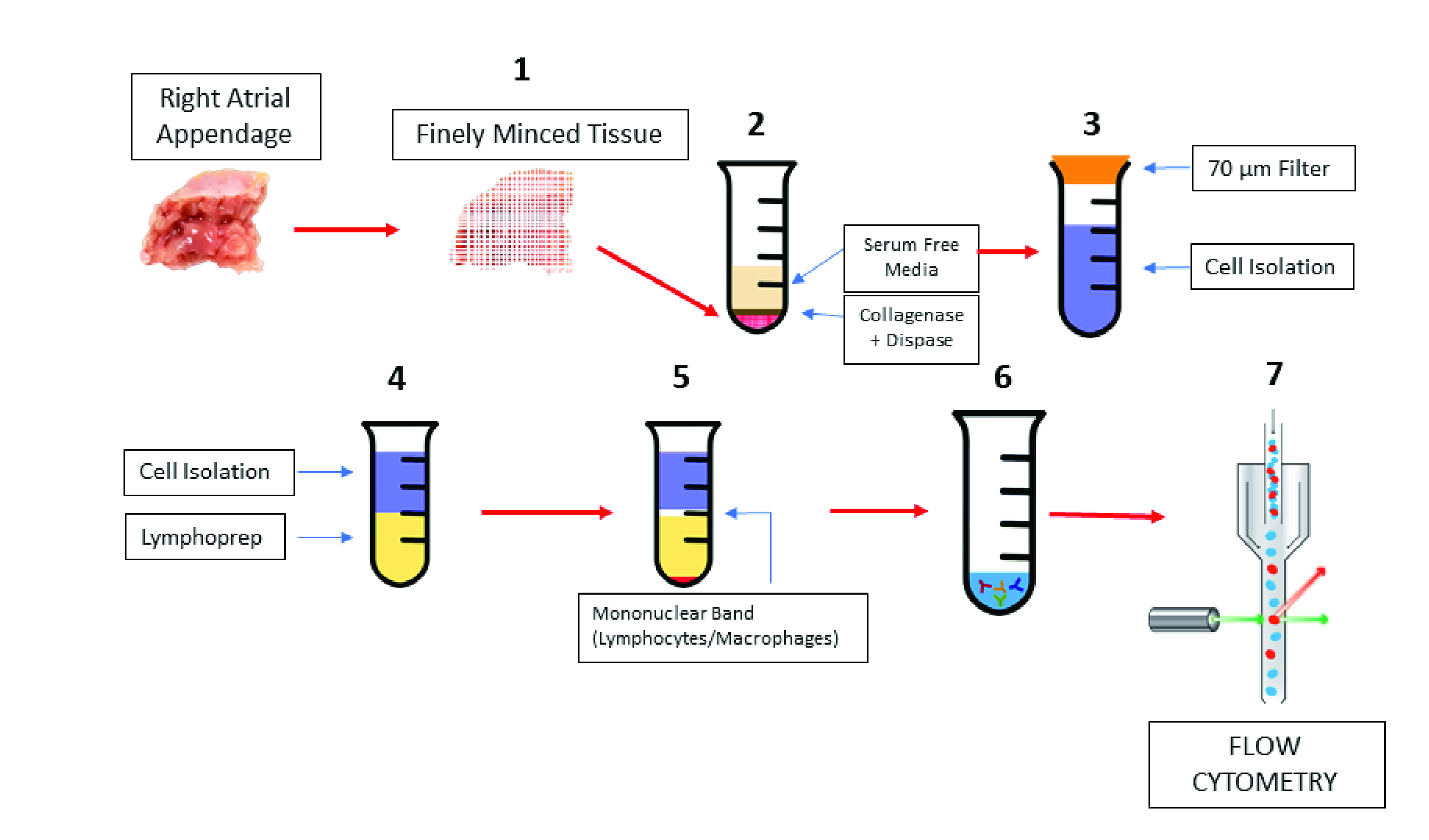

Supplement: Supplementary file 1 — Additional file 1: Figure S1. (1) The right atrial appendage is finely minced and (2) treated with collagenase and dispase in serum free HBBS media. (3) The cells are then filtered through a 70um filter and washed twice to isolate cells. (4) Cells are isolated over Lymphoprep and centrifuged for 30 min at 400G with no brake. (5) Mononuclear cells at the interphase are isolated and (6) incubated with anti-human antibodies linked to different fluorescent labels for (7) flow cytometry analysis. [file 12967_2019_2162_MOESM1_ESM.tif]

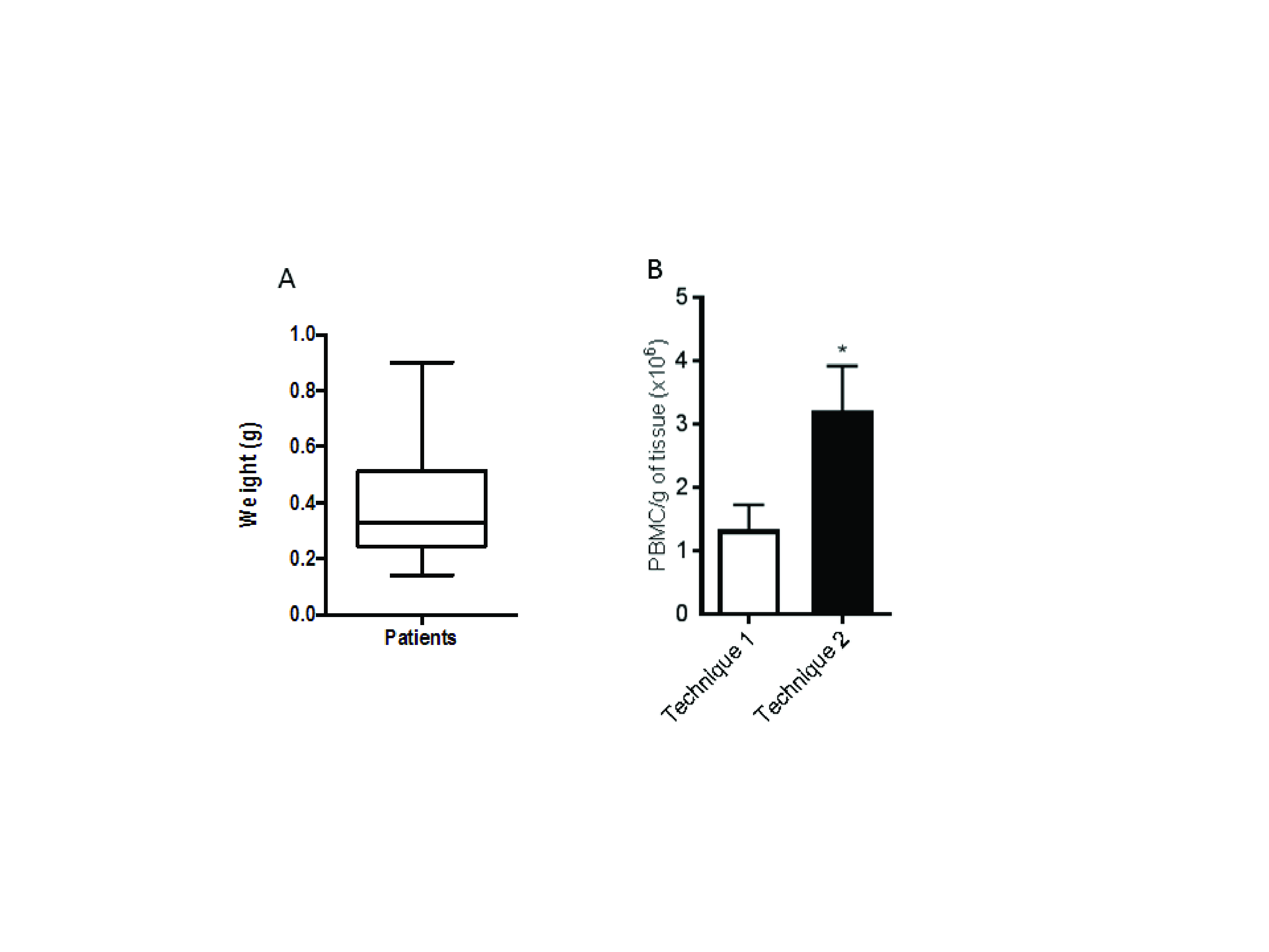

Supplement: Supplementary file 2 — Additional file 2: Figure S2. Macrophage isolation purity. (A) Dot plot representing the variability of obtainable tissue weight. (B) PBMC isolation comparison between two different techniques. Technique 1: incubation of tissue with collagenase for 30 min. Technique 2: incubation of tissue with collagenase + dispase for 45 min. Technique 2 yielded a higher cell isolation that was significantly different from technique 1 (P = 0.02). [file 12967_2019_2162_MOESM2_ESM.tif]

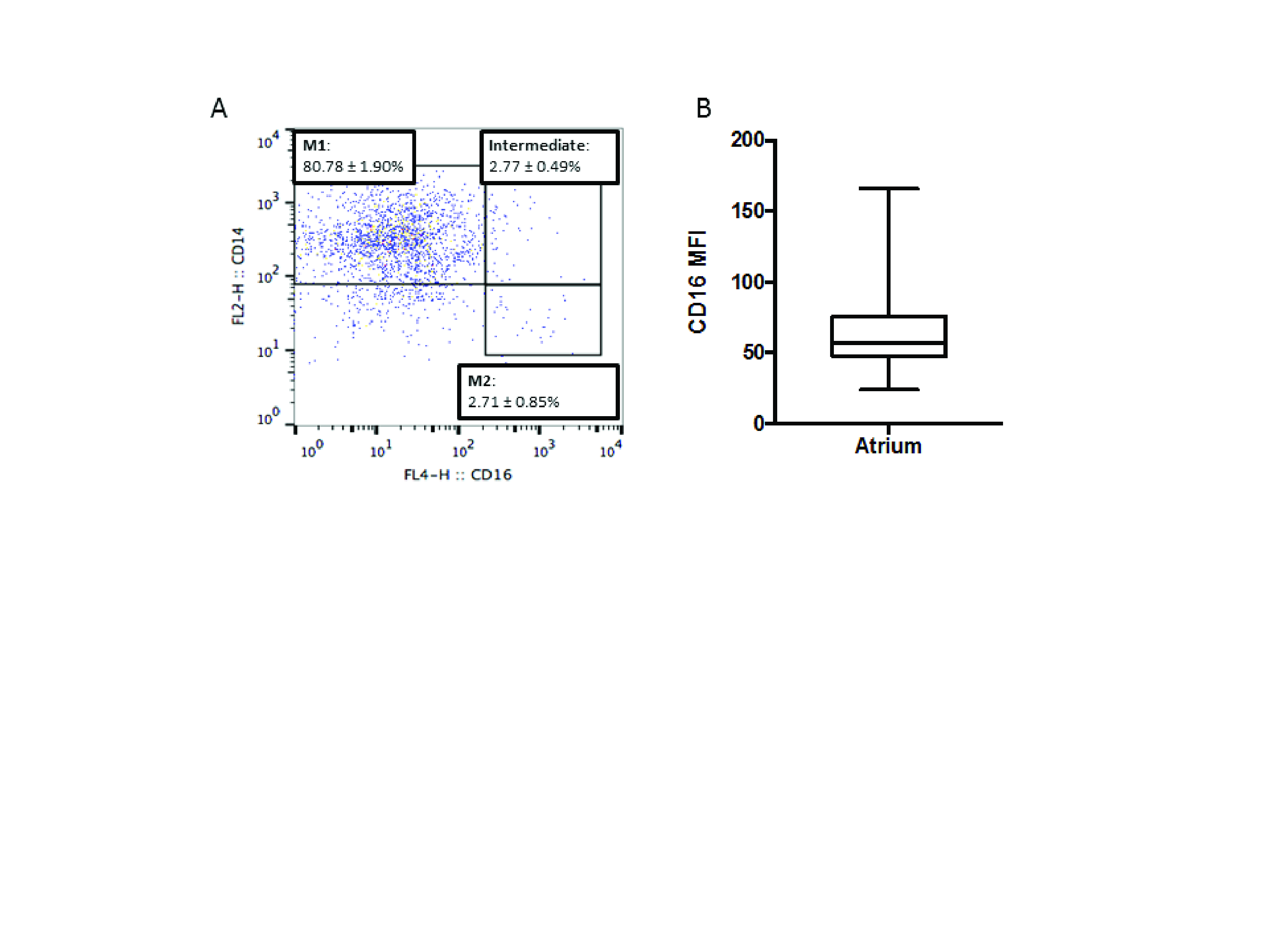

Supplement: Supplementary file 3 — Additional file 3: Figure S3. Flow cytometry macrophage subset analysis. (A) Flow cytometry dot plot representing gated macrophage populations (CD16 x CD14) as a mean value (n = 18). (B) CD16 MFI values in atrial tissue isolates. [file 12967_2019_2162_MOESM3_ESM.tif]

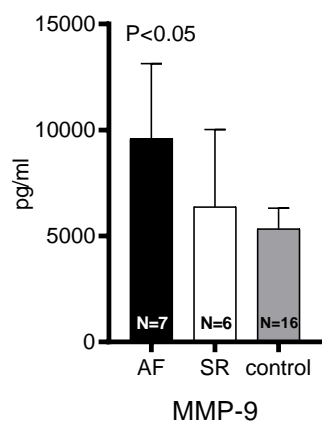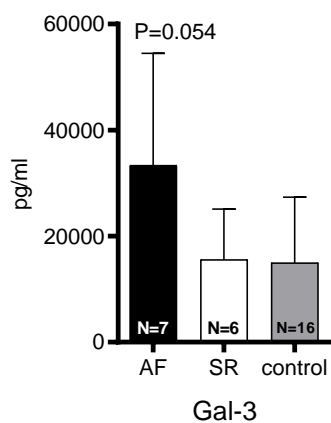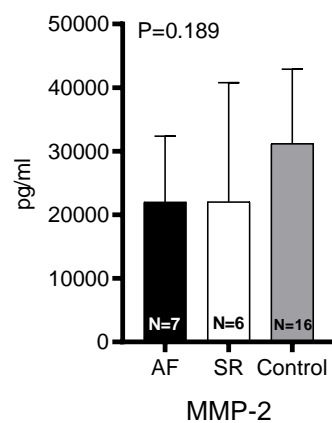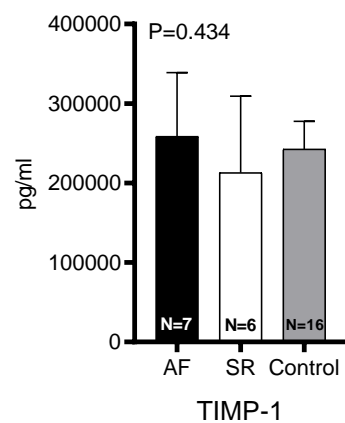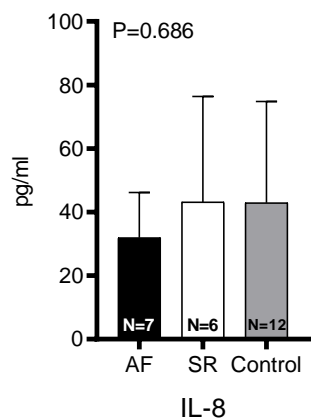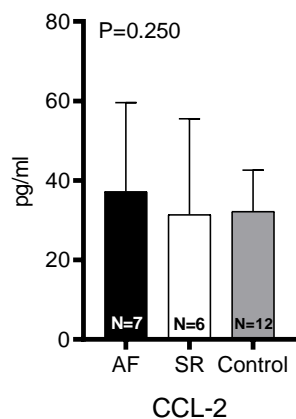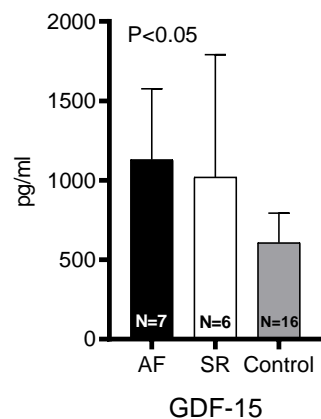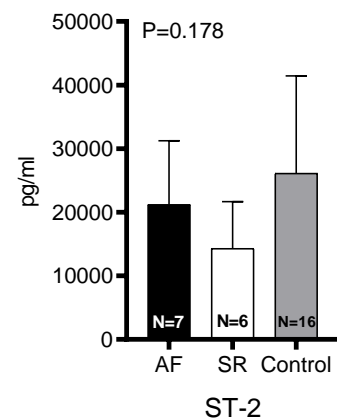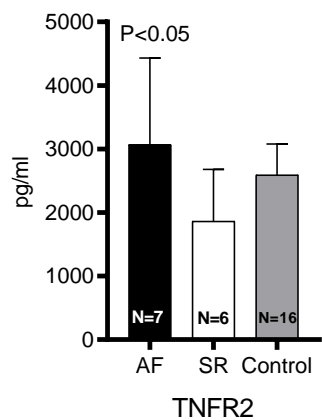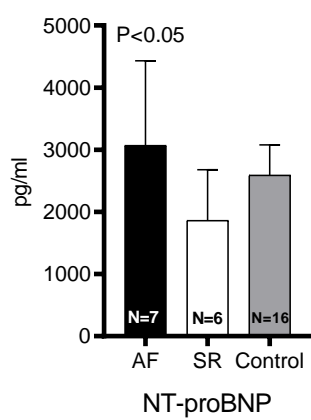

Supplement: Supplementary file 4 — Additional file 4: Figure S4. Luminex analyses of cytokines in AFib, SR and control patients. Cytokines that were significantly elevated in AFib patients were GDF-15, TNFR2, NT-propBNP. [file 12967_2019_2162_MOESM4_ESM.pdf]
